# Joint Preservation in Acetabular Subchondral Cyst Management: A Case Report

**DOI:** 10.7759/cureus.82092

**Published:** 2025-04-11

**Authors:** Alkisti Konstantinou, Sotiris Plakoutsis, Christos Konstantinidis, Dimitrios Vardakas, Dimitrios Giotis

**Affiliations:** 1 Department of Orthopaedic Surgery, General Hospital of Ioannina "G. Hatzikosta", Ioannina, GRC

**Keywords:** acetabular cyst, curettage, excision, hip joint, joint preservation, management

## Abstract

The purpose of this study is to present the management of a symptomatic acetabular subchondral cyst in the right hip in the absence of significant osteoarthritis, along with the joint-preserving surgical approach undertaken. We highlight the efficacy of open surgical excision and bone grafting in relieving symptoms and preserving the integrity of the native hip joint. This case report details a patient with a two-year history of progressive gait impairment and localized pain in the right hip. Radiographic and advanced imaging revealed a subchondral cyst in the right acetabular sourcil without significant evidence of osteoarthritis. After a thorough evaluation, the patient underwent excisional curettage of the cyst followed by the implantation of a cancellous allogenic bone graft. Postoperatively, the patient experienced rapid symptom relief, significant functional improvement, and a return to daily activities without pain. By demonstrating successful functional recovery and pain resolution without the need for total hip arthroplasty, this study aims to contribute to the existing literature on alternative treatment strategies for acetabular subchondral cysts, particularly in patients without advanced joint degeneration.

## Introduction

Subchondral bone cysts are fluid-filled cavities that develop beneath the articular cartilage, most commonly in weight-bearing joints such as the hip, knee, and shoulder [1]. They are predominantly associated with degenerative joint conditions, particularly osteoarthritis (OA) [1]. Other contributing factors include joint trauma, instability, metabolic bone disorders such as avascular necrosis, and inflammatory arthropathies like rheumatoid arthritis [1].

The exact pathogenesis of subchondral cysts remains under investigation, but two primary theories are widely accepted. The first is the synovial fluid intrusion theory, suggesting that joint fluid penetrates through microscopic defects in the cartilage into the subchondral bone, resulting in cyst formation. The second is the bone necrosis theory, which proposes that mechanical stress leads to localized subchondral bone necrosis followed by cystic degeneration [1-4]. Histological studies commonly reveal fibrous tissue lining, bone remodeling, and the presence of osteoclasts and osteoblasts, supporting the degenerative and reactive nature of these lesions [2].

Radiologically, OA is characterized by various radiological findings, including multiple small cysts, subchondral sclerosis, osteophytes, intra-articular osteochondral bodies, and narrowing or complete loss of joint space [1]. Acetabular subchondral cysts are frequently observed in patients with OA and are closely linked to disease progression [1].

Subchondral cysts are typically asymptomatic and are most often detected incidentally through radiological imaging during OA evaluation [1]. However, in some cases, they can cause pain, localized swelling, joint stiffness, and an increased risk of fracture due to weakened bone structure [1]. While there is no standardized classification system specifically for assessing fracture risk in subchondral cysts, factors such as cyst size, location in weight-bearing zones, and the extent of cortical bone thinning can contribute to an increased risk and guide clinical decision-making [1]. A subset of patients with acetabular subchondral cysts may experience greater pain, increased disability, and reduced functional capacity [1].

The initial diagnosis is usually made through plain radiographs. Once a lesion is identified, further imaging with computed tomography (CT) scans or magnetic resonance imaging (MRI) is often performed for a more detailed assessment [3]. The management of subchondral bone cysts in the acetabulum depends on factors such as cyst size, symptom severity, and the extent of joint involvement [4]. Asymptomatic cases are typically monitored with periodic imaging, while patients with mild symptoms often benefit from conservative treatment, including physical therapy, non-steroidal anti-inflammatory drugs (NSAIDs), or intra-articular corticosteroid injections for symptomatic relief [3]. In cases of advanced OA, total hip arthroplasty (THA) remains the gold standard for restoring function and relieving pain [1-4].

While acetabular subchondral cysts are frequently encountered in the setting of advanced OA, their presence in the absence of joint space narrowing poses unique diagnostic and therapeutic challenges [1,3]. In such cases, especially in younger or functionally active patients, THA may be considered premature [4]. Instead, joint-preserving strategies can offer substantial clinical benefits, including pain relief, functional improvement, and delay of arthroplasty [3]. Understanding when and how to apply such approaches is clinically important, as it supports the long-term preservation of the native joint and maintains the quality of life in a patient population that may otherwise face early prosthetic intervention [4]. However, the literature remains limited on the surgical management of isolated symptomatic acetabular cysts without concurrent OA, which underscores the relevance of reporting such cases [1].

The purpose of this study is to present the joint-preserving management of a female patient with a large symptomatic subchondral cyst in the acetabular sourcil, which involved open surgical excision and curettage of the cyst, followed by bone graft placement. We highlight the importance of individualized, joint-preserving surgical strategies in managing symptomatic acetabular cysts, particularly when joint replacement is not yet indicated.

## Case presentation

A 63-year-old female patient presented to our hospital with a two-year history of progressive gait impairment. Over the two-year period, the patient experienced a gradual increase in right hip pain, described as a deep, aching discomfort that worsened with prolonged standing, walking, or climbing stairs. The pain was most prominent in the groin region and occasionally radiated to the anterior thigh. She reported morning stiffness lasting around 10-15 minutes and noted difficulty rising from a seated position. Functional limitations progressively increased, ultimately requiring the use of a walking cane for ambulation. These symptoms significantly impacted her ability to perform daily activities, including household tasks and recreational walking. Her medical history was unremarkable, with no prior trauma or systemic pathology. Physical examination revealed tenderness during the range of motion (ROM) assessment of the right hip, along with a clearly noticeable gait disturbance. Compared to the contralateral side, the right hip showed a reduced ROM, which affected her ability to walk for extended periods or rise from seated positions without support. Her Harris Hip Score (HHS) was 68, and pain intensity was 7 on the Visual Analog Scale (VAS). Systemic and extra-articular causes of hip pain, including lumbar spine pathology (e.g., herniated disc and spinal stenosis), sacroiliac joint dysfunction, ischiofemoral impingement, and rotational deformities, were considered and clinically excluded based on a thorough physical examination and correlating imaging findings.

**Figure 1:**
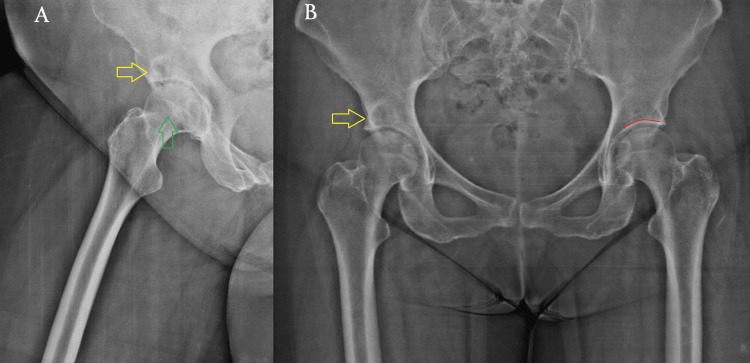
Preoperative X-rays. The yellow arrows indicate a well-defined subchondral cyst located in the weight-bearing region of the acetabular sourcil. No significant joint space narrowing or osteophyte formation is seen (Tönnis Grade 1). (A) Right hip. The green arrow shows the femoral head. (B) Pelvis. The curved red line outlines the acetabular sourcil (roof) of the contralateral hip joint.

**Figure 2:**
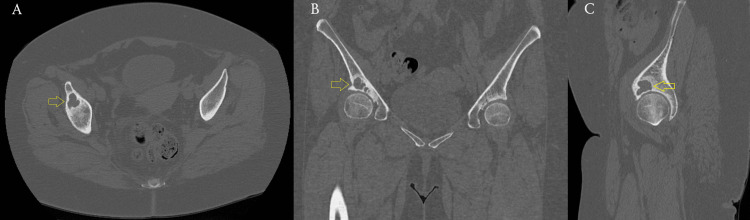
Preoperative CT scan. (A) Axial plane. (B) Coronal plane. (C) Sagittal plane. Yellow arrows demonstrate a multiloculated cystic lesion within the subchondral bone of the acetabular dome, confirming its location in the weight-bearing region. CT: computed tomography

**Figure 3:**
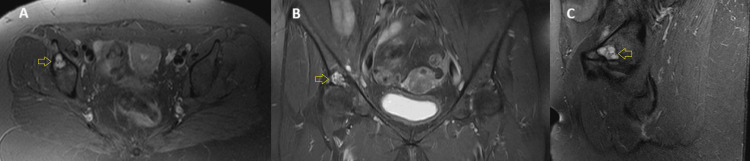
Preoperative MRI. Axial (A), coronal (B), and sagittal (C) T2-weighted sequences reveal a hyperintense lesion (yellow arrows), consistent with a fluid-filled subchondral cyst. No labral tear, cartilage delamination, or joint effusion is noted. MRI: magnetic resonance imaging

A radiographic evaluation, including standard anteroposterior and lateral hip views, was performed, revealing a well-defined subchondral cyst in the right acetabular sourcil (Figure 1). Although there were no signs of advanced degenerative changes, radiographic assessment showed slight joint space narrowing without significant osteophyte formation or subchondral sclerosis, corresponding to Tönnis Grade 1, which is consistent with early-stage OA. To further evaluate the lesion, advanced imaging with CT and MRI was conducted (Figures 2, 3). The cyst was well-demarcated, located within the weight-bearing region of the right acetabulum, and measured 2.3 × 1.9 cm. MRI evaluation revealed no evidence of labral tear, capsular injury, or articular cartilage surface defect, and there were no signs of intra-articular loose bodies or effusion. Given the absence of compelling radiological evidence of OA that would typically warrant total hip replacement (THR), a joint-preserving surgical approach was pursued.

**Figure 4:**
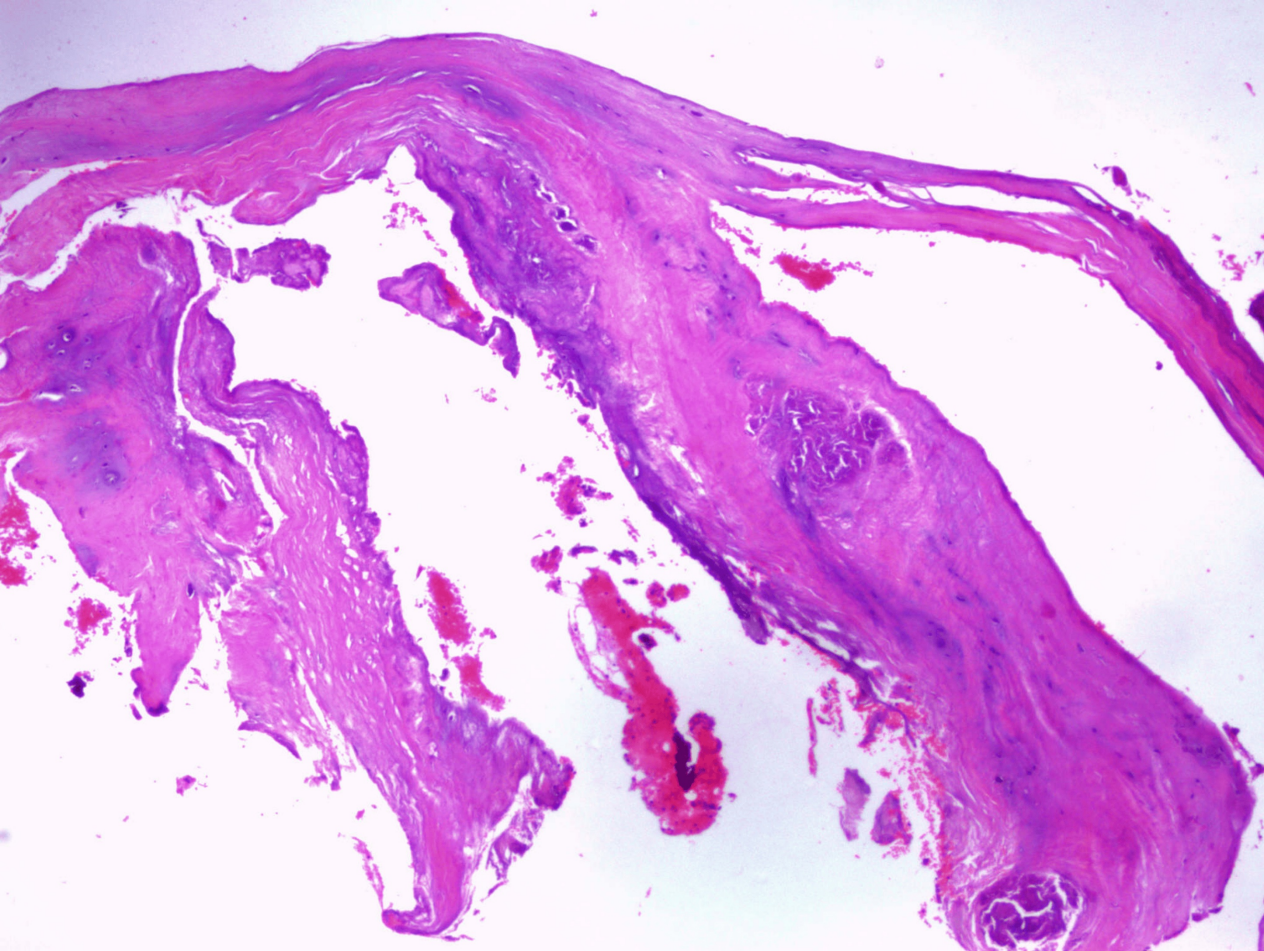
Histopathological image from the biopsy of the surrounding cystic capsule, stained with hematoxylin and eosin (H&E). The wall is composed of fibrous connective tissue with areas of myxoid degeneration, confirming the diagnosis of a benign subchondral cyst.

The patient underwent open surgical excision and curettage of the subchondral cyst, followed by allogenic bone grafting to restore acetabular integrity. An anterior open surgical approach was utilized, involving an osteotomy of the anterior inferior iliac spine (AIIS) to access the acetabular roof. This step provided improved visualization of the weight-bearing region of the acetabulum, where the cyst was located, allowing for complete excisional curettage. Once access was established, the subchondral cyst was identified and fully exposed. Curettage was performed to remove all cystic contents, ensuring complete debridement of the lesion. There was no communication between the cyst and the acetabular labrum, and no paralabral cysts were observed intraoperatively. Following cyst excision, 10 cc of cancellous allogenic bone graft was implanted into the defect to restore acetabular integrity and facilitate osseous healing. A biopsy of the surrounding cystic capsule was obtained and sent for histopathological examination, which confirmed the diagnosis of a subchondral cyst (Figure 4). Finally, osteosynthesis was performed using a 3.5-mm titanium cortical screw with a washer to secure the osteotomy site and ensure structural stability.

**Figure 5:**
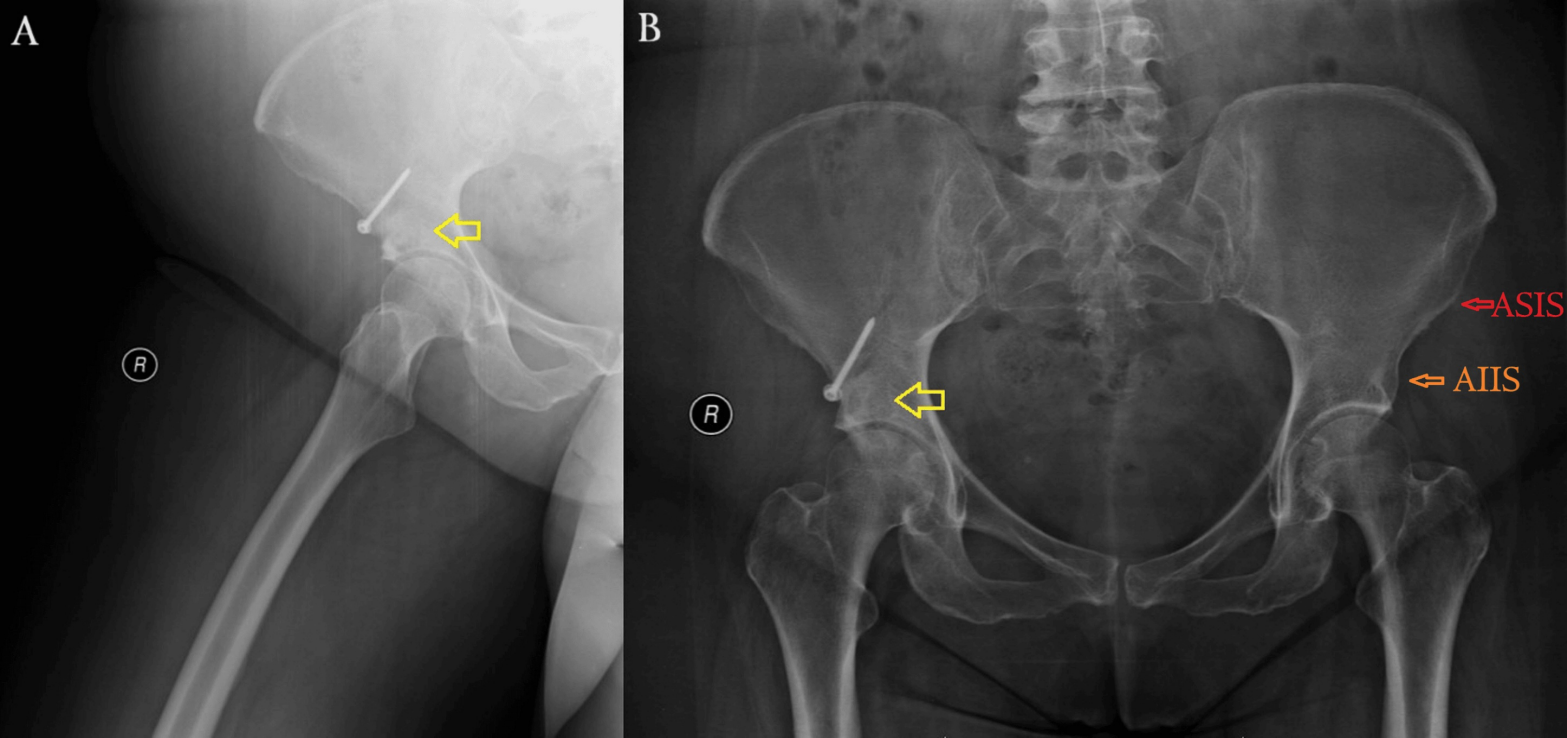
X-rays at three months post-op showing radiographic signs of osseous healing (marked by yellow arrows). (A) Right hip. (B) Pelvis. A single 3.5-mm cortical screw is seen securing the osteotomy at the AIIS. Satisfactory graft placement and alignment are noted. ASIS: anterior superior iliac spine; AIIS: anterior inferior iliac spine

**Figure 6:**
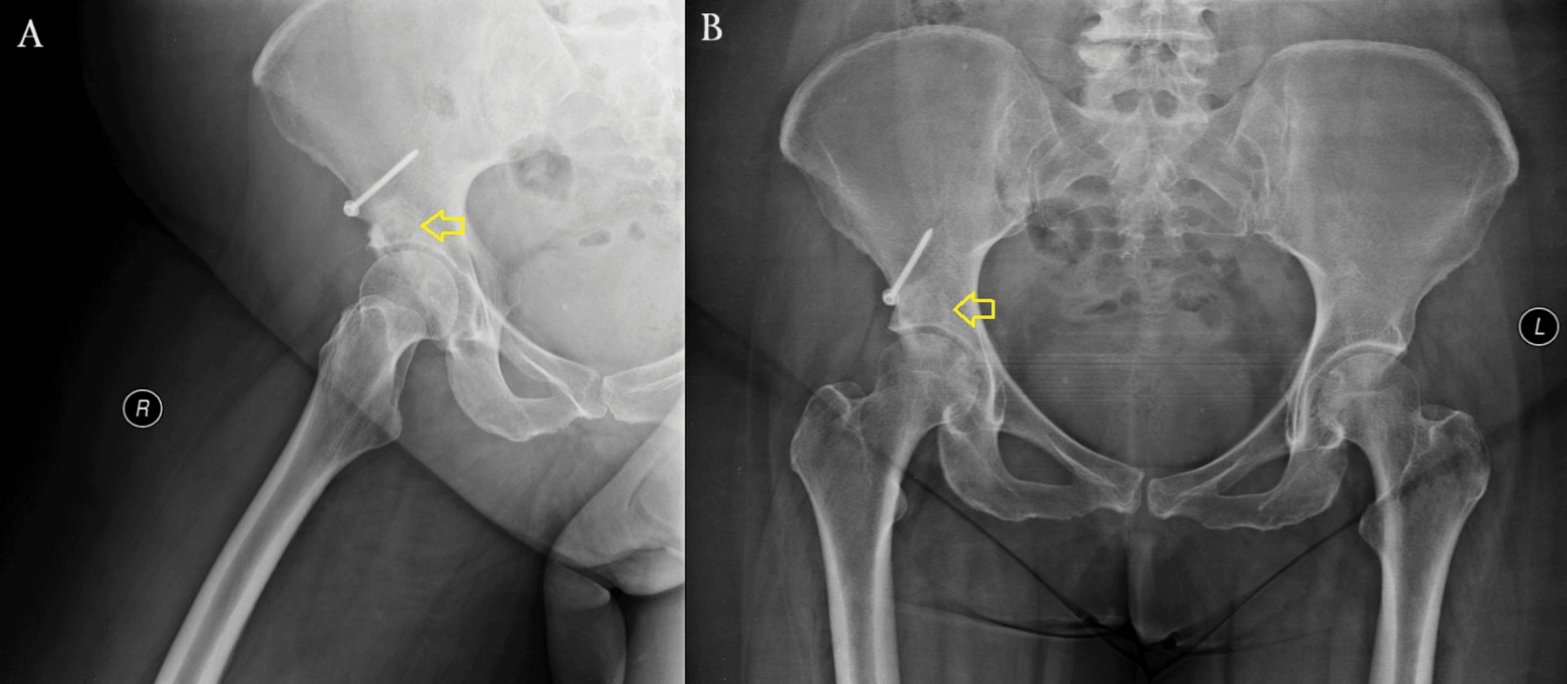
X-rays at six months post-op demonstrating further graft incorporation (pointed out by yellow arrows). (A) Right hip. (B) Pelvis. No signs of collapse or cyst recurrence are seen.

**Figure 7:**
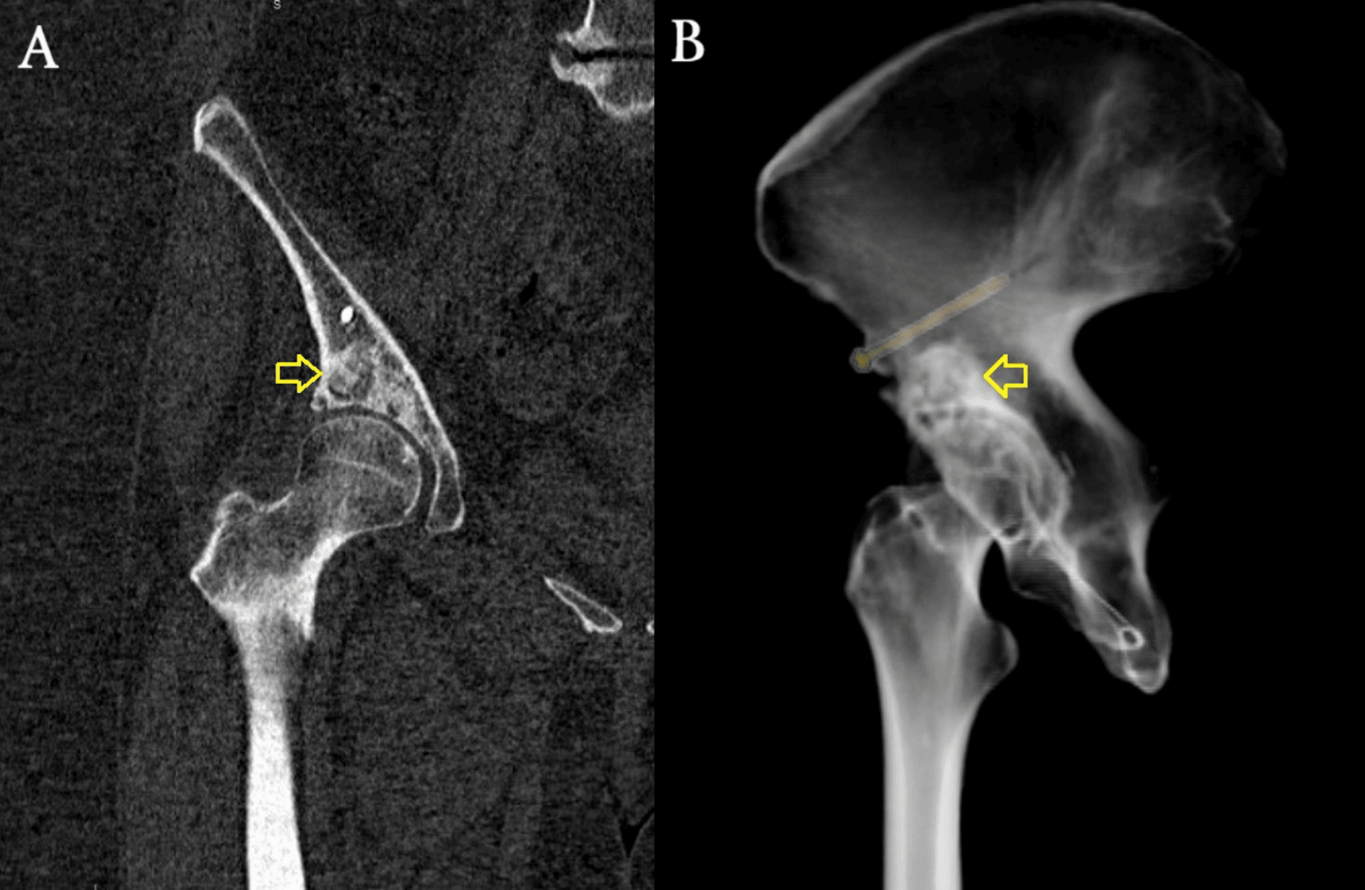
CT at six months post-op. Continued bone graft consolidation is visible (yellow arrows). No joint space narrowing or new cystic lesions are noted. (A) Coronal view. (B) 3D reconstruction. CT: computed tomography

**Figure 8:**
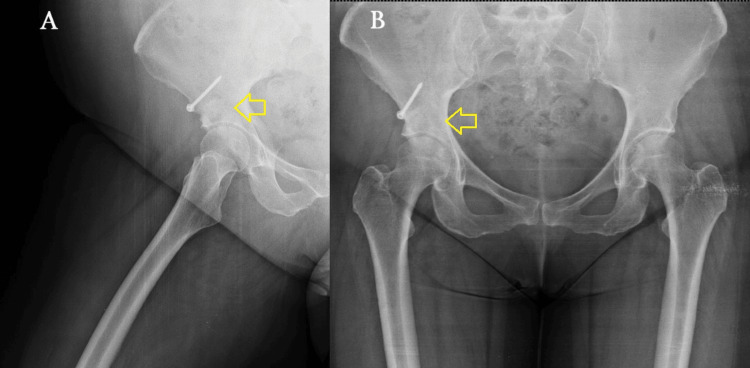
X-rays at one year post-op showing complete graft incorporation (pointed out by yellow arrows). There is no radiographic evidence of osteoarthritic progression or recurrence of the lesion. (A) Right hip. (B) Pelvis.

**Figure 9:**
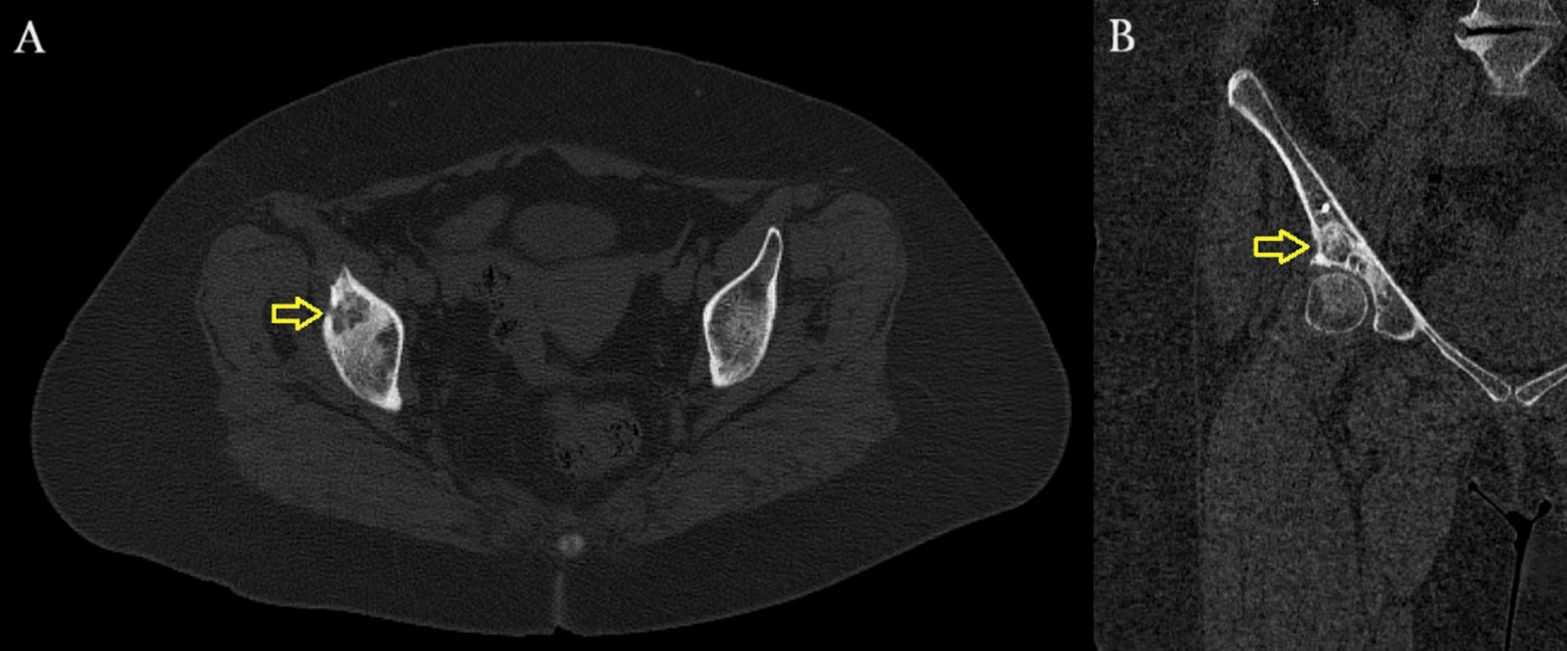
CT at one year post-op. The yellow arrows show the remodeled acetabular bone at the former cyst site. No signs of residual cyst or osteolysis are observed. (A) Transverse view. (B) Coronal view. CT: computed tomography

In the initial postoperative (post-op) period, the patient ambulated with crutches under a non-weight-bearing protocol for the first two weeks. Passive and active-assisted hip ROM exercises were recommended to minimize stress on the joint. From the third week onward, a gradual transition to partial weight-bearing was allowed as tolerated, depending on pain levels. At 12 weeks post-op, after radiographic signs of graft incorporation, the patient progressed to full weight-bearing without an assistive device (Figure 5). By four months post-op, she had returned to her daily activities without pain, presenting only a slight residual deficiency in ROM (Figures 6, 7). At the one-year follow-up, she remains pain-free without any progression of OA changes in her right hip joint (Figures 8, 9). Her HHS improved from 68 preoperatively to 90, indicating an excellent functional outcome with substantial improvement in hip mobility and quality of life. In parallel, her pain intensity on the VAS decreased from 7 preoperatively to 0, further supporting the effectiveness of the joint-preserving surgical approach (Table 1).

**Table 1:** Chronological timeline of clinical events and interventions. This timeline illustrates the patient’s clinical course from the initial onset of right hip pain through progressive symptomatology, diagnosis, surgical intervention, rehabilitation, and recovery. CT: computed tomography; MRI: magnetic resonance imaging

Timepoint	Clinical event/intervention
-26 months	Onset of mild right hip pain
-20 months	Worsening gait disturbance-began using a walking aid
-2 months	Presentation to the hospital with progressive symptoms
-1 month	Radiographic evaluation with X-ray, CT, and MRI
0 point	Open surgical excision of cyst and bone graft placement
+2 weeks	Initiation of physiotherapy under non-weight-bearing protocol
+12 weeks	Full weight-bearing resumed without assistive devices
+4 months	Return to daily activities
+12 months	Pain-free, no further progression of right hip osteoarthritis, excellent functional outcome

The patient provided written informed consent for the publication of this case report, including all relevant clinical information and images.

## Discussion

Subchondral cysts in the acetabular region are commonly found in weight-bearing areas and are often associated with OA [1,5,6]. While these cysts are typically asymptomatic, in some cases, they can cause pain, joint stiffness, and functional limitations, significantly affecting a patient’s quality of life [1,6,7]. When symptomatic, subchondral cysts may require surgical intervention, especially in cases where joint integrity is at risk or conservative treatments fail [8].

In advanced hip OA, subchondral cysts in the acetabular sourcil are frequently present, often leading to the necessity of THR. THR remains the gold standard for patients with significant joint degeneration, as it effectively relieves pain, improves mobility, and restores function [6]. However, in cases where hip OA is not yet advanced, but the presence of a symptomatic cyst impairs function, a joint-preserving approach should be considered [3,8].

Various surgical techniques for treating acetabular subchondral cysts have been reported, ranging from arthroscopic debridement to open surgical excision with bone grafting [3,4,6,9,10]. The key advantage of these surgical strategies is their ability to relieve symptoms while preserving the native hip joint. Additionally, they often allow for a rapid recovery, enabling patients to resume daily activities within a short period [3]. In our patient, given the absence of significant OA changes, a joint-preserving approach was selected to address the symptomatic subchondral cyst in the acetabular sourcil.

While arthroscopic techniques offer the benefit of minimally invasive access and quicker rehabilitation, their feasibility depends heavily on cyst size, location, and accessibility. Arthroscopic debridement and bone grafting of deep acetabular cysts may be effective when cysts are accessible and not extensively large [4,9,10]. Marty et al. demonstrated favorable outcomes using arthroscopic bone grafting in deep acetabular cysts [3]. Similarly, Garabekyan et al. presented a technique using a curved delivery device to reach and fill cysts arthroscopically, suggesting good accessibility in selected patients [9].

In our case, the cyst measured 2.3 × 1.9 cm and was located in the central weight-bearing region of the acetabular sourcil, a zone that is difficult to visualize and access arthroscopically, even for experienced hip arthroscopists [8]. Furthermore, to reach the cyst arthroscopically, the intact overlying cartilage would need to be disrupted, thereby creating an iatrogenic chondral defect. This compromises the integrity of the articular surface and may not be effectively repaired through arthroscopy, ultimately contradicting the aim of joint preservation. In contrast, the open approach via AIIS osteotomy enabled direct visualization, complete curettage, controlled graft impaction, and, importantly, preservation of the overlying cartilage, which remained structurally intact. In parallel, osteosynthesis with a 3.5-mm titanium cortical screw provided structural stability, allowing for controlled post-op rehabilitation.

Despite arthroscopic advancements in treating such cases, open surgical excision with bone grafting still plays an important role for cysts of increased size or those located in anatomically challenging regions, as it provides full exposure, allows for complete debridement, and ensures accurate graft placement [1,10]. This approach is particularly valuable when the overlying cartilage needs to be directly visualized and preserved [1,10].

Although bone grafting is an effective strategy to restore structural integrity after cyst curettage, it carries potential risks such as graft incorporation failure, graft resorption, infection, and donor site morbidity (in cases of autografting) [11,12]. Graft incorporation failure may result from inadequate mechanical stability, insufficient host bone vascularity, or incomplete cyst debridement [11,12]. To mitigate these risks, meticulous curettage of the cyst, selection of a high-quality cancellous allogenic graft, and rigid fixation of the osteotomy site were employed.

The patient’s post-op course followed a structured weight-bearing progression, ensuring proper healing of the bone graft and minimizing joint stress. Significant symptom relief was achieved post-op with the patient returning to his daily activities after a few months. At the one-year follow-up, she remained pain-free with excellent functional recovery and showed no signs of OA progression. Notably, preserving the native hip joint contributed to a faster and more efficient rehabilitation process.

The management of acetabular subchondral cysts requires a balance between symptom relief and joint preservation. While cyst excision is essential to prevent further joint deterioration and pain, maintaining the structural and functional integrity of the hip is paramount. Preserving the native hip joint helps maintain natural biomechanics and leads to better long-term outcomes by reducing the risk of joint degeneration, ultimately delaying or preventing the need for more invasive procedures, such as THA [6]. Additionally, joint-preserving techniques may contribute to lower healthcare costs, as they can postpone the need for hip replacement, which is often associated with prolonged recovery periods and higher medical expenses [4,6,13].

This case report has several limitations that should be acknowledged. First, it represents a single patient, which limits the generalizability of the findings to broader populations. Second, the follow-up period of one year, while encouraging, may not be sufficient to assess long-term outcomes such as cyst recurrence and progressive joint degeneration. Third, no quantitative gait or biomechanical analysis was performed, and the assessment of functional recovery relied primarily on clinical scores and patient-reported outcomes.

## Conclusions

In conclusion, the management of subchondral cysts in the acetabular sourcil through excisional curettage and bone grafting provides an effective approach for symptom relief and functional restoration, especially in the absence of advanced OA. By preserving the integrity of the native hip joint, this procedure provides satisfactory long-term functional outcomes and reduces the need for more invasive interventions such as THA. However, as this report is based on a single case with limited follow-up, broader generalizations should be made with caution. Further studies involving larger patient cohorts and long-term follow-up are necessary to confirm the durability and reproducibility of these results.
